# 

**Published:** 1871-05

**Authors:** 


MAY, 1871.

### THE AMERICAN JOURNAL OF DENTAL SCIENCE,

EDITED BY

F. J. S. GORGAS, M. D., D. D. S.

PUBLISHED MONTHLY.

$2.50 PER ANNUM.

VOL. 5.—THIRD SERIES.—NO. 1.

BALTIMORE:

SNOWDEN & COWMAN.

LONDON:

TRUBNER & CO., 60 PATERNOSTER ROW.

1871.

PAYABLE IN ADVANCE.

